# Integrated analysis of single-cell and bulk transcriptomics develops a robust neuroendocrine cell-intrinsic signature to predict prostate cancer progression

**DOI:** 10.7150/thno.92336

**Published:** 2024-01-01

**Authors:** Tingting Zhang, Faming Zhao, Yahang Lin, Mingsheng Liu, Hongqing Zhou, Fengzhen Cui, Yang Jin, Liang Chen, Xia Sheng

**Affiliations:** 1 Key Laboratory of Environmental Health, Ministry of Education & Ministry of Environmental Protection, School of Public Health, Tongji Medical College, Huazhong University of Science and Technology, Wuhan, China.; 2 School of Life and Health Sciences, Hainan University, Haikou, China.; 3 Department of Neurology, Wuhan Fourth Hospital/Pu'ai Hospital, Wuhan, China.; 4 The Second Ward of Urology, Qujing Affiliated Hospital of Kunming Medical University, Qujing, China.; 5 Institute for Cancer Genetics and Informatics, Oslo University Hospital, Oslo, Norway.; 6 Department of Urology, Tongji Hospital, Tongji Medical College, Huazhong University of Science and Technology, Wuhan, China.

**Keywords:** neuroendocrine prostate cancer, single-cell, biomarker, computational biology and bioinformatics, machine learning

## Abstract

Neuroendocrine prostate cancer (NEPC) typically implies severe lethality and limited treatment options. The precise identification of NEPC cells holds paramount significance for both research and clinical applications, yet valid NEPC biomarker remains to be defined.

**Methods:** Leveraging 11 published NE-related gene sets, 11 single-cell RNA-sequencing (scRNA-seq) cohorts, 15 bulk transcriptomic cohorts, and 13 experimental models of prostate cancer (PCa), we employed multiple advanced algorithms to construct and validate a robust NEPC risk prediction model.

**Results:** Through the compilation of a comprehensive scRNA-seq reference atlas (comprising a total of 210,879 single cells, including 66 tumor samples) from 9 multicenter datasets of PCa, we observed inconsistent and inefficient performance among the 11 published NE gene sets. Therefore, we developed an integrative analysis pipeline, identifying 762 high-quality NE markers. Subsequently, we derived the NE cell-intrinsic gene signature, and developed an R package named NEPAL, to predict NEPC risk scores. By applying to multiple independent validation datasets, NEPAL consistently and accurately assigned NE feature and delineated PCa progression. Intriguingly, NEPAL demonstrated predictive capabilities for prognosis and therapy responsiveness, as well as the identification of potential epigenetic drivers of NEPC.

**Conclusion:** The present study furnishes a valuable tool for the identification of NEPC and the monitoring of PCa progression through transcriptomic profiles obtained from both bulk and single-cell sources.

## Introduction

Prostate cancer (PCa) is the second most common cancer in male affecting millions of men worldwide 1, 2. Androgen receptor (AR) signaling plays a central role in PCa progression, while targeting the AR pathway can lead to profound response in hormone sensitive PCa (HSPC). Unfortunately, the disease often recurs into a more aggressive phenotype known as the castration-resistant PCa (CRPC), most of which is still histologically classified as adenocarcinoma (CRPC-Adeno) with reactivated AR pathway 1, 3. Of note, approximately 17% of CRPC displays neuroendocrine (NE) phenotype (CRPC-NE) to different extent, and may further develop to the poorly differentiated NE PCa (NEPC), a subtype generally implies severe lethality and lack of therapeutic option 1, 4-6.

The prevalence of NEPC is anticipated to increase as patients undergo multiple lines of treatments 1. NE tumor cells can be histologically distinguished from other cells residing in the complex PCa microenvironment (TME); but their scarcity, in particular in early stage of the disease 7, leads to frequent missed diagnosis of early NEPC. Currently, NEPC diagnosis mainly depends on the immunohistochemistry of several biomarkers - negative AR, high MKI67, and positive NE markers, such as CHGA, SYP, ENO2 and NCAM1 8, 9. However, these proteins are heterogeneously expressed in NE tumor cells, which greatly impairs their diagnostic sensitivity 10.

The advent of next-generation sequencing endows comprehensive depiction of the molecular landscape of NEPC. Critical drivers of NEPC have been established, such as mutations in *FOXA1* and *SPOP* in primary PCa, lineage plasticity induced by *RB1* loss and *TP53* dysfunction, as well as activation of the polycomb-repressive complex-2 (PRC2) such as EZH2 in advanced PCa 4, 5, 9, 11, 12. Meanwhile, these studies have proposed more than 10 NEPC related gene sets, encompassing thousands of differentially expressed genes (DEGs) collectively. However, these gene sets are of considerable heterogeneity, to which possible reasons include: a. most of these studies were based on limited number of NEPC cases 13; b. the gene expression profiles between CRPC-Adeno and NEPC are surprisingly similar 14; c. these gene sets relied heavily on transcriptomic data derived from bulk tumors instead of NE tumor cells exclusively 15. Therefore, there remains an urgent need to develop sensitive and specific NEPC markers for the purposes of both basic research and clinical translation.

In this study, we first assembled a large scRNA-seq meta-atlas of human PCa, and revealed the poor consistency and weak power of the 11 published NE gene sets. To generate a better NEPC predictor, we then developed an integrative pipeline combining bulk transcriptomic data, scRNA-seq data and multiple algorithms, identified 771 high-quality NEPC feature markers and a NE cell-intrinsic gene signature, and constructed a robust NEPC risk prediction model. Using numerous datasets derived from both human PCa cohorts and experimental models of PCa, we showed that our NEPC classifier displayed remarkable power in predicting disease progression to NEPC, prognosis, and therapy responsiveness, which outperformed all published PCa prognostic models. Thus, our model offers a useful reference for precise identification and characterization of NE tumor cells.

## Materials and Methods

### Single-cell RNA-seq data processing

In this study, we collected and analyzed a total of 11 scRNA-seq datasets (9 as discovery datasets and the other 2 as validation datasets) covering primary HSPC (Pri), CRPC, mCRPC and NEPC 10, 15-21 (Table S1). Each of these datasets has undergone individual quality control and pre-annotation analysis by us. Potential doublets predicted by Scrublet in Python (v.3.8.5), low quality cells (cells with <250 detected genes, <500 transcripts, or >20% mitochondrial content) and high dropout genes were removed to avoid interference with the analysis. Besides, to avoid unexpected noise and expression artefacts by dissociation, genes associated with mitochondria (50 genes) and ribosome (1,253 genes) were excluded. After pre-annotation and merging, 210,879 high-quality cells with 20,870 genes from 66 tumor samples (scRNA-seq meta-atlas) were preserved for subsequent analyses. Seurat (v4.1.0) or Scanpy (v.1.9.1) was then applied to perform normalization, identification of variably expressed genes, and principal component analysis. To mitigate the batch effects from utilization of diverse platforms and protocols, we employed 11 classical integration algorithms alongside an unintegrated method as a reference, including BBKNN, CCA, RPCA, LIGER, Harmony, fastMNN, scVI, scANVI, Scanorama, Connos and Combat. Then clustering and distribution of single cells is mapped using Uniform Manifold Approximation and Projection (UMAP) with the Leiden algorithm. Based on pre-annotation labels and scib evaluation 22, we determined that the BBKNN method was more suitable for our scRNA-seq meta-atlas, thereby being chosen for further analysis. Finally, a total of 15 cell types was identified by manual annotation (corresponding biomarkers, see Table S1) and combined automatic annotation method Celltypist 23. For NE tumor cells subclustering analysis, a similar procedure was applied.

Seurat (v.4.1.0) was applied for subsequent analyses and visualization. To investigate interconversion and evolutionary trajectories of different NE cell types, we applied the Monocle 3 (https://cole-trapnell-lab.github.io/monocle3/) and CytoTRACE 24 algorithms according to demonstration notebook.

### Bulk dataset selection and preparation

In this study, we assembled over 3000 samples from 15 publicly available human bulk PCa datasets. As previously described 25, The Cancer Genome Atlas Prostate Adenocarcinoma (TCGA-PRAD) 11 RNA-seq data (Raw read count) was downloaded from the UCSC XENA. International Cancer Genome Consortium (ICGC) PRAD RNA-seq data was downloaded from the ICGC portal (https://dcc.icgc.org/). RNA-seq data for SU2C 5, WCM 4, and MCTP 26 datasets were downloaded from the cBio Cancer Genomics Portal (https://www.cbioportal.org/). RNA-seq data for WCDT 27 was downloaded from http://davidquigley.com/prostate.html. RNA-seq data for CPGEA 28 was downloaded from www.cpgea.com. RNA-seq data for PCaProfiler 29 was downloaded from www.PCaProfiler.com. The microarray data including MSKCC 30, CamCap 31, UM/SPORE 26, GSE54460 32, GSE116918 33 and GSE84042 34, and RNA-seq data for UW/RA 35 were acquired from the Gene Expression Omnibus (GEO, https://www.ncbi.nlm.nih.gov).

Besides, we collected transcriptomic data for 13 experimental models of PCa, including 8 human PCa cell lines (from The Cancer Cell Line Encyclopedia, CCLE), two patient-derived xenografts (PDX) models (UW/RA and GSE199596 36) and three genetically engineered mouse models (GSE69903 37, GSE90891 12, and OncoLoop 38). For all RNA-seq data, Transcript Per Million (TPM) value was calculated. To note, due to the distinct dynamic ranges and batch characteristics between bulk RNA-seq and microarray data, and in order to ensure a fair evaluation of our model, we analyzed the aforementioned bulk datasets separately, instead of conducting an integrative analysis.

### Spatial transcriptomic data processing

Spatial transcriptome sequencing data (GSE230282 39) was downloaded from GEO (Table S1). Pathologists have annotated this tissue as NEPC coexisting with HSPC. Spatial transcriptome sequencing data was standardized and corrected using the Sctransform method according to corresponding tutorial manual of Seurat (https://satijalab.org/seurat/articles/spatial_vignette.html).

### Meta-analysis of published NEPC gene lists

We searched the literature for published NE gene sets from differentially expressed analyses between NEPC and prostatic adenocarcinoma based on transcription profiling of patient tumor samples (Table ​S2). Besides, HP_NE_neoplasm gene set representative pan-NE neoplasm was obtained from MSigDB database (http://www.gsea-msigdb.org/). To compare different gene sets and identify common genes, gene names and probe assignments were updated to hg38 HGNC symbols.

### Enrichment analysis

As previously described 40, the GO and rank-based gene set enrichment (GSEA) analyses were performed by the R package clusterProfiler. For bulk transcriptomic data, single-sample GSEA (ssGSEA) method was conducted using R package GSVA. To overcome dropouts and technical variation of scRNA-seq, we chose AUCell algorithm for scRNA-seq data to optimize the discovery and characterization of cell states 41. R package “estimate” was used to evaluate the stromal score, immune score and tumor purity of each samples using bulk transcription profile data 42.

### Analysis of the tumor mutation status in the low- and high- NEPC risk groups

The somatic mutation data was downloaded from the Genomic Data Commons Data Portal (https://portal.gdc.cancer.gov/) for TCGA Primary PCa samples, and cBioPortal (https://www.cbioportal.org/) for SU2C mCRPC database. Concerning different mutation types, including frame shift del, frame shift ins, in frame del, in frame ins, missense, nonsense, nonstop, splice site and translation start site were regarded as nonsynonymous mutation variants 40. Silent and other mutation types, including 3' flank, 5' flank, 3' UTR, 5'UTR, RNA, IGR, intron, and splice region, were treated as synonymous mutations, which was regarded as a wild type 40. The mutation landscape for different groups was exhibited using maftools R package.

### Development and validation of NEPC prediction model

We designed a pipeline (illustrated in Figure 2A) to construct a NEPC prediction model. First, we acquired 588 validated NE markers by scRNA-seq meta-atlas from 1482 meta-NE markers. Meanwhile, using WGCNA method based on PCaProfiler bulk RNA-seq data, we identified two potential NE related gene modules. Moreover, pseudobulk differential expression analysis was applied for scRNA-seq meta-data, which led to the identification of 1896 NEPC differentially expressed genes (DEGs). Next, combined with the results, we identified 587 up-regulated and 184 down-regulated high-quality NEPC feature genes, termed NE_FG (Table S3). Last, seven classical machine learning algorithms were performed to construct a NEPC prediction model based on the NE_FG expression profiles, including elastic network (Enet), least absolute shrinkage and selection operator (LASSO), ridge regression, gradient boosting machine (GBM), random forest (RSF), supervised principal components (SuperPC), and support vector machine (SVM). In addition, we also identified two thinned NE cell-intrinsic gene signatures by overlapping the NE_FG and DEGs for NE tumor cells by FindMarkers function using wilcoxon rank sum test with Bonferroni correction (|avg_log2FC|>= 0.5, p_val_adj < 0.01) in scRNA-seq meta-atlas, termed NE_UP (n = 90) and NE_DN (n = 40) signature. ssGSEA algorithm was then applied and NE_UP_DN risk score for each sample in each cohort was defined as follows:



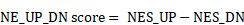



Where NES_UP was the ssGSEA score for NE_UP gene signature, and NES_DN was the ssGSEA score for NE_DN gene signature. Finally, in 6 validation cohorts containing NEPC tumors (SU2C, WCDT, PCaProfiler, UM/SPORE, WCM and scRNA-seq Psedobulk cohorts), we calculated NEPC risk scores for each model (see Table S4). Models were evaluated by three scRNA-seq cohorts and indexes including C-index, receiver-operator characteristic (ROC) curve, correlation analysis and area under the curve (AUC).

All algorithms, models, published NE gene sets, and NEPC signature identified in this study were packaged as Neuroendocrine Prostate Cancer Algorithms (NEPAL) R package (https://github.com/Famingzhao/NEPAL) for NEPC risk calculation, which was validated for both human and mouse transcriptomic expression profiles. Note that we have removed GBM, SuperPC, and RSF models considering the overfitting.

### TF activity inference

For bulk transcriptomic data, transcription factor activity for each sample was inferred using the VIPER package 43. The TF targets were collected from DoRothEA and the medium confidence targets were used for analysis.

### Statistical analysis

All data analyses were performed in the R (v.4.1.0) or Python (v.3.8.5) platform. The Student's t-test or Wilcoxon rank-sum test was applied to compare continuous variables between two groups, while one-way ANOVA or Kruskal-Wallis tests were used to conduct difference comparisons of three groups. Correlations between normally distributed variables were performed with Pearson's correlation, and correlations between non-normally distributed variables were assessed with Spearman's correlation. The Benjamini-Hochberg (BH) method was introduced to estimate false discovery rate for multiple testing. Kaplan Meier analysis with log-rank tests was performed to assess survival difference between groups via “survminer” R package. P-value < 0.05 was considered statistically significant with two sides. Odds ratios (ORs), hazard ratios (HRs) and 95% confidence intervals (CIs) were reported if necessary.

## Results

### Previous NEPC gene sets display low consistency and poor power

First, we collected and analyzed all 11 published NE marker gene sets, including 9 NEPC gene-lists from bulk transcriptomic data, 1 from scRNA-seq of normal prostate, and 1 representative pan-NE neoplasm from the MSigDB database (Table S2). Collectively, these 11 gene sets consisted of 1482 up-regulated expression NE markers (NEPC_Meta). However, a low overlap rate was observed among these gene sets, with only 61 genes overlapping more than four times (Figure S1A).

**Figure 1:**
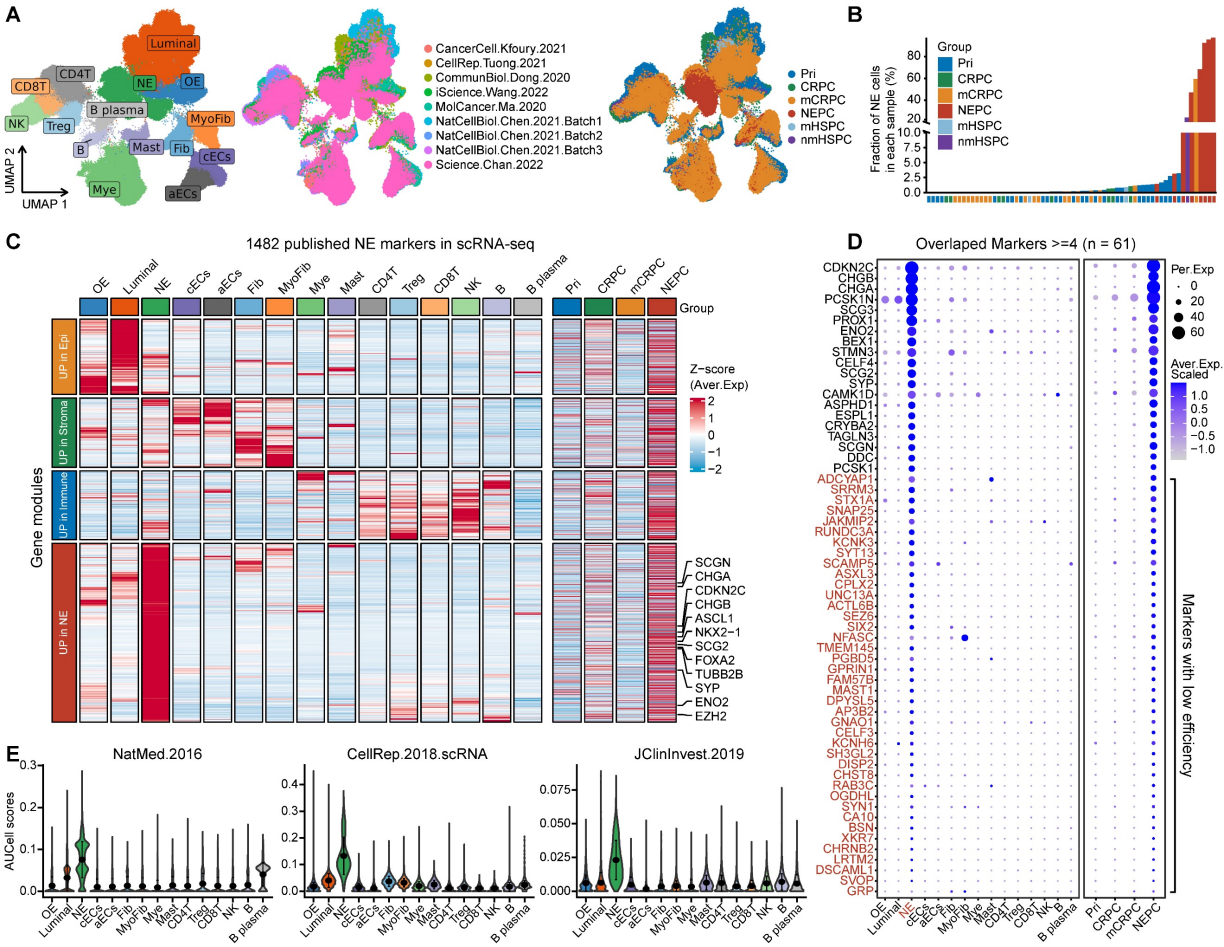
** scRNA-seq analysis revealed the poor sensitivity and low efficiency of the published NEPC gene sets. A.** The UMAP plot of 210,879 single cells from 66 PCa samples in scRNA-seq meta-atlas. Left, 16 major cell types; middle, 9 published data sources; right, different tumor subtypes. **B.** The distribution of estimated NE tumor cells among different groups. **C.** Heatmap showing the expression (Z-score) of 1482 published NE markers in different cell types (left panel) and tumor types (right panel). **D.** Dot plot of high overlap NE marker genes (n = 61) from meta-gene sets for each cell cluster and tumor group. **E.** AUCell enrichment analysis comparing different NE gene sets in each cell type.

To evaluate the sensitivity and efficiency of these NE markers, we generated a comprehensive scRNA-seq reference atlas with a total of 210,879 single cells from 66 PCa tumors covering primary HSPC (Pri), CRPC, mCRPC and NEPC, based on 9 published human PCa scRNA-seq datasets (Figure 1A and Table S1; see method). A total of 15 cell types were identified by corresponding biomarkers (Table S1), and frequency of NE tumor cells for each sample was then calculated (Figure 1B). Surprisingly, we found that more than half of these NE markers (894/1482) were not exclusively expressed in NE tumor cells or in patients with NE features (Figure 1C and Figure S1B). As for the above-mentioned 61 genes with high overlap rate, despite that they could well discern NEPC tumors, more than half (41/61) exhibited low percentage expressive abundance (percentage expression for all NE tumor cells < 20%) (Figure 1D), implicative of their weak efficiency. Lastly, by calculating the NE score for each gene sets using AUCell enrichment analysis 41, we confirmed the low specificity of most gene sets to recognize NE tumor cells in scRNA-seq data (Figure 1E and Figure S1C). These results revealed low consistency and poor power of the published NE gene sets.

### Construction of NEPC classifier based on large scRNA-seq and bulk RNA-seq meta-databases

**Figure 2:**
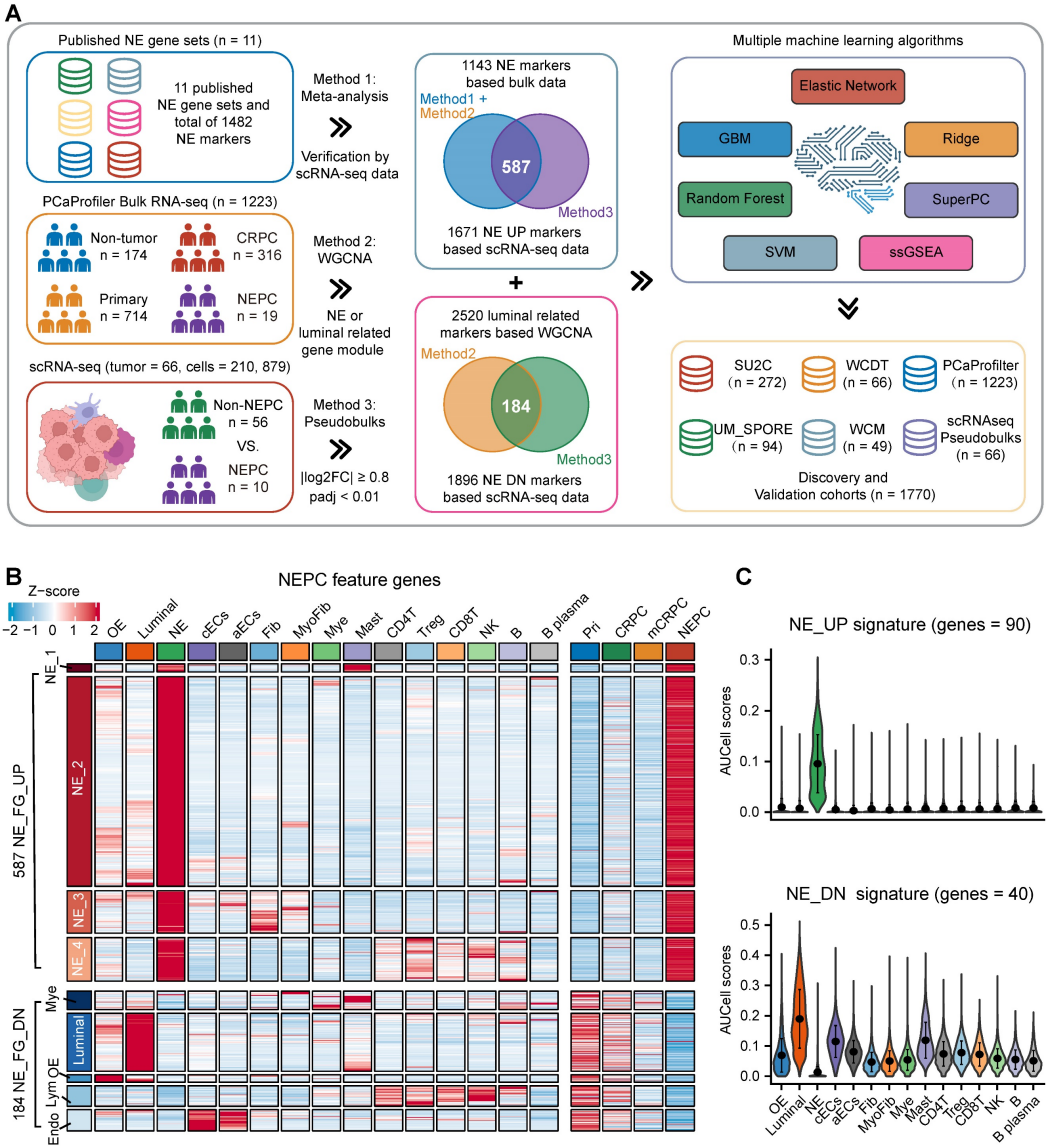
** Combining multiple strategies to identify NEPC markers based on scRNA-seq and bulk RNA-seq meta-databases. A.** Strategic schema about identification of high-quality NEPC markers and construction of predictors via multiple machine learning algorithms. Elastic network, Enet. Ridge regression, Ridge. Gradient boosting machine, GBM. Random forest, RSF. Supervised principal components, SuperPC. Support vector machine, SVM. **B.** Heatmap showing the expression (Z-score) of 587 up-regulated and 184 down-regulated NEPC feature genes (NE_FG) identified by this study. **C.** Violin plot showing the thinned up- (upper panel) and down-regulated (lower panel) NEPC signature by AUCell analysis.

To identify high-quality NEPC feature markers, we designed a pipeline comprising published NEPC_Meta markers as mentioned above, WGCNA gene modules based on the bulk RNA-seq dataset PCaProfiler (n = 1223, Figure S2A), and the PCa scRNA-seq meta-atlas we assembled (Figure 2A, see methods). Finally, 587 up-regulated and 184 down-regulated NEPC feature genes were identified, which were collectively termed as NE_FG (Figure 2B and Table S3). Since signatures focused on cancer-cell intrinsic gene expression were found to more clinically useful 44, 45, we also obtained two thinned NE cell-intrinsic gene signatures, termed NE_UP (n = 90) and NE_DN (n = 40), by overlapping the NE_FG with DEGs of NE tumor cells (Figure 2C and Table S3; see methods). Dot plot confirmed that all NE_UP signature genes had high percentage expressive abundance (percentage expression for all NE tumor cells > 20%; Figure S2B).

**Figure 3:**
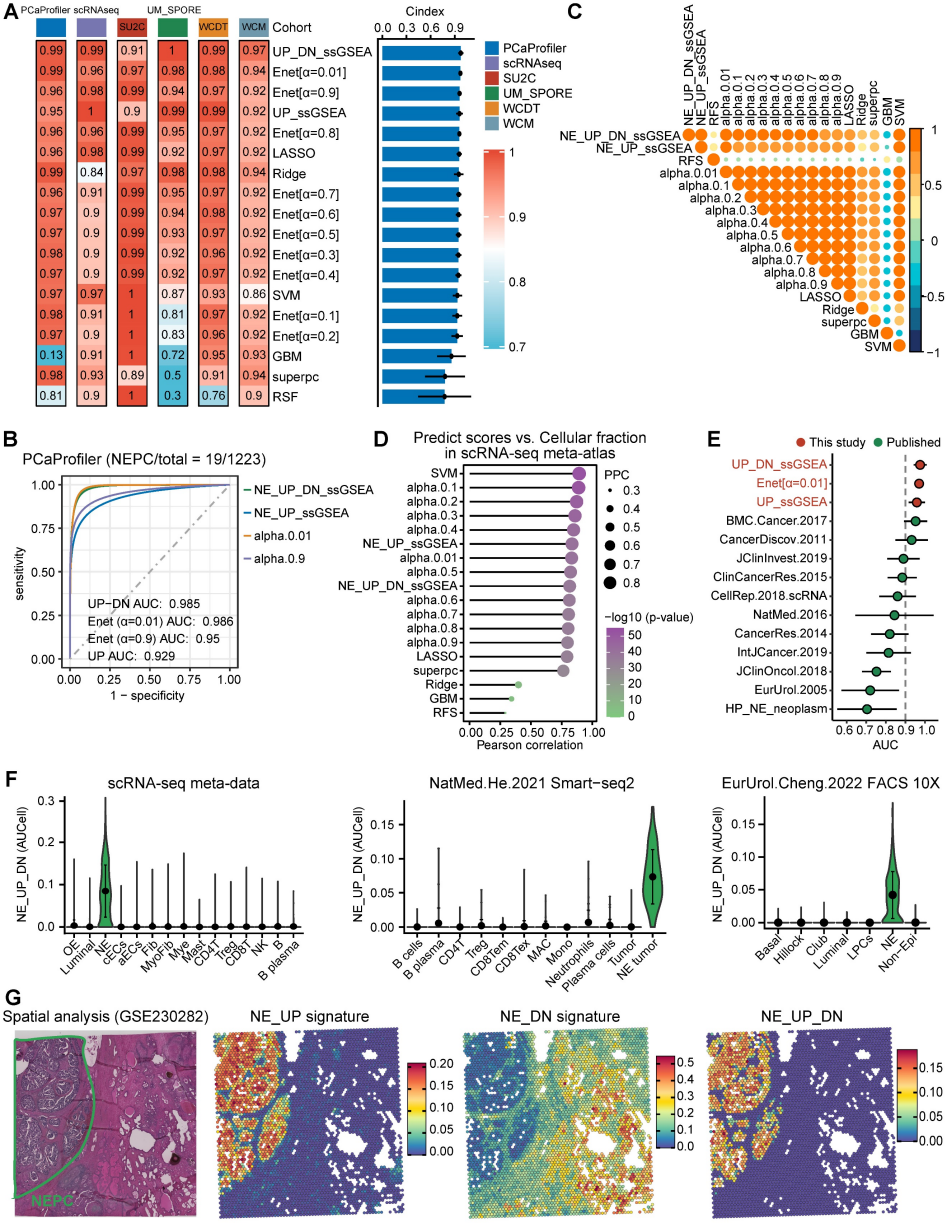
** Construction and validation of the artificial intelligence-derived NEPC risk prediction models. A.** The C-indexes of 18 algorithms in the 6 validation cohorts. Error bar denote SD. **B.** Time-dependent receiver-operator characteristic (ROC) analysis for predicting NEPC in PCaProfiler bulk RNA-seq cohort (n = 1223). **C.** Heatmap showing the correlation analysis of NEPC risk scores estimated by different models in PCaProfiler. **D.** Correlation analysis between cellular fraction of NE tumor cells from scRNA-seq meta-atlas and different NEPC risk algorithms. PCC, Pearson correlation coefficient. **E.** Comparing AUC index of the top 3 predictors from this study and 11 published NE gene sets in 6 cohorts. Error bar denote SD. **F.** Violin plot showing NEPC risk scores estimated by NE_UP_DN model with AUCell algorithm for each cell type in scRNA-seq meta-atlas and two independent scRNA-seq datasets. Error bar denote SD. **G.** H&E staining and heatmaps of the spatial distribution of NE_UP, NE_DN or NE_UP_DN signature in multiple regions.

To further construct a NEPC prediction model, we applied 7 classic machine learning algorithms based on NE_FG to SU2C training set. Besides, NE_UP_DN model combining NE_UP and NE_DN was constructed based on ssGSEA algorithm (see methods). Subsequently, using these NEPC predictors, we calculated NEPC risk score for each sample in 6 cohorts containing NEPC tumors. For the evaluation index, we calculated the average C-index (Figure 3A) and R^2^ (Figure S2C) for each algorithm. Interestingly, most of these predictors had a high average C-index, which may be attributed, at least in part, to our high-quality NE markers. Of these models, NE_UP_DN_ssGSEA, Enet [α= 0.01], and NE_UP_ssGSEA ranked as top 3, which also had high area under the ROC curves (AUCs > 0.90, Figure 3B). In addition, most predictors, except RSF and GBM models, showed high Pearson correlation coefficients for NEPC prediction scores (Figure 3C). Taking advantage of the scRNA-seq meta-atlas, we found that most algorithms again showed high Pearson correlation coefficient between predicted NEPC risk scores and cellular fraction of NE tumor cells (Figure 3D). Meanwhile, we compared our models with the 11 published NEPC_Meta gene sets by calculating AUCs index using the six validation datasets, where our top 3 models unanimously outperformed the previous NE gene-lists (Figure 3E).

For validation, we selected the best classifier, NE_UP_DN signature, and evaluated its predictive performance in the scRNA-seq meta-atlas and three additional validation scRNA-seq and spatial transcriptomic datasets: a. Smart-seq2 based scRNA-seq dataset 16, b. single-cell dataset based on fluorescence-activated cell sorting (FACS) 20, c. spatial gene expression atlas of de novo NEPC coexisting HSPC 39. The results showed that NE_UP_DN with AUCell algorithm precisely predicted NEPC cell status in all validation sets (Figure 3F-G). Taken together, these results demonstrate that our models could robustly distinguish tumors with NE features based on transcriptomic data of both bulk and single-cell sources. Of note, we performed subsequent analyses using the NE_UP_DN signature, which we hereafter referred to as NEPC algorithm (NEPAL).

### NEPAL to portrait the path of PCa progression

**Figure 4:**
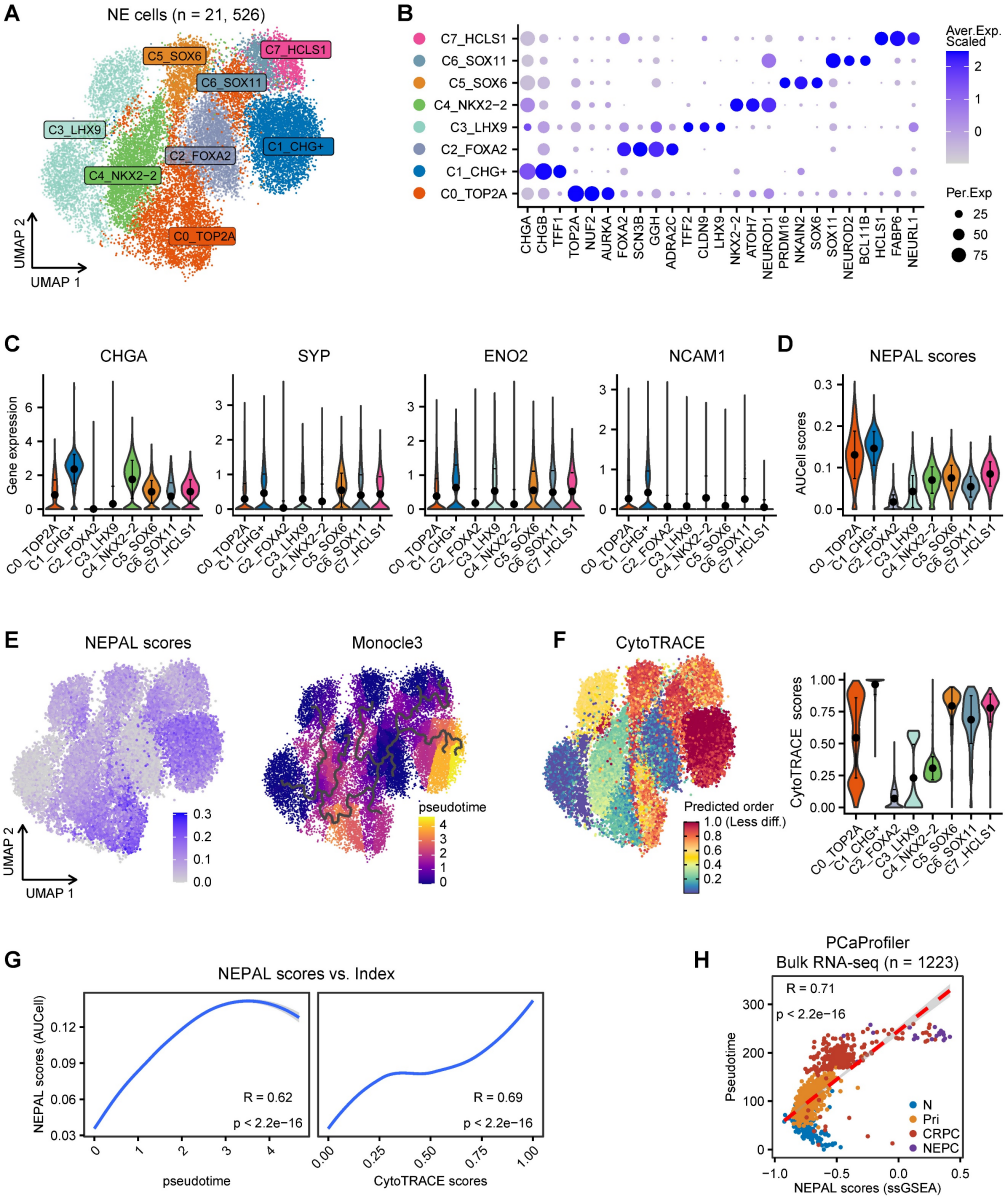
** NEPAL to portrait the path of PCa progression. A.** The UMAP plot showing 8 NEPC subclusters from 21,526 single cells based on scRNA-seq meta-atlas. **B.** Dot plot of representative marker genes for each NEPC subcluster. **C.** Violin plot showing the expression of classic NE markers for each cell type. Error bar denote SD. **D.** AUCell analysis of NEPAL signature among 8 NEPC subclusters. **E.** The distribution of NEPAL risk scores (left panel) and Pseudotime analysis of NEPC subclusters inferred by Monocle3 (right panel). **F.** CytoTRACE analysis for differentiation status of each cell type. **G.** Pearson correlation analysis between NEPAL risk scores and pseudotime index (left panel) or CytoTRACE scores (right panel) in NEPC single cells. **H.** Scatter diagram showing relationship between NEPAL risk scores and pseudotime in PCaProfiler large bulk RNA-seq cohort.

In addition to distinguishing NEPC, we hypothesized that NEPAL could quantify NEPC progression, as it incorporates both upregulated and downregulated NE cell-intrinsic signature genes into the model. To assess this hypothesis, we first re-clustered 21,526 NE tumor cells from the scRNA-seq meta-atlas, which led to the identification of 8 general NEPC subclusters (Figure 4A-B). Concordant with a recent report 8, classic NE markers such as *CHGA*, *SYP*, *ENO2* and *NCAM1* were heterogeneously expressed amongst these subclusters (Figure 4C). Importantly and by contrast, NEPAL with AUCell algorithm was almost ubiquitously expressed in all NEPC subclusters (Figure 4D). Next, pseudo-time and CytoTRACE analyses were performed on the 8 NEPC subclusters (Figure 4E-F), and the evolutionary trajectory was highly correlated with the NEPAL risk scores (Figure 4G), suggesting its utility in predicting NEPC progression.

Intriguingly, we found marked correlation between NEPAL risk indices and pseudotime scores in PCaProfiler (Figure 4H). Also, we examined the relationship between NEPAL risk indices and Gleason scores in four independent datasets, TCGA PRAD, CamCap, ICGC PRAD, and CPGEA, where concordant and significant correlation was observed in primary tumors without castration or NE features (Figure S3A). Collectively, these results implicate that NEPAL can be applied to predict disease progression for PCa of both hormone-sensitive and -refractory stages.

### Application of NEPAL to experimental models of PCa

To validate its utility, we further applied NEPAL to the transcriptomic profiles derived from experimental models of PCa. For the 8 human PCa cell lines from CCLE, NEPAL accurately assigned the NEPC cell line NCHI-H660 with the highest NEPC risk score. CRPC cell lines, such as DU145, 22RV1, and PC3 cells, followed closely behind (Figure S3B), while the hormone-dependent cell lines, such as MDA-PCa-2B and LNCaP, had the lowest NEPC risk scores. Besides, we observed a high Pearson correlation coefficient between NEPAL scores and NE classic markers, such as *CHGA* and *SYP* (Figure S3B).

**Figure 5:**
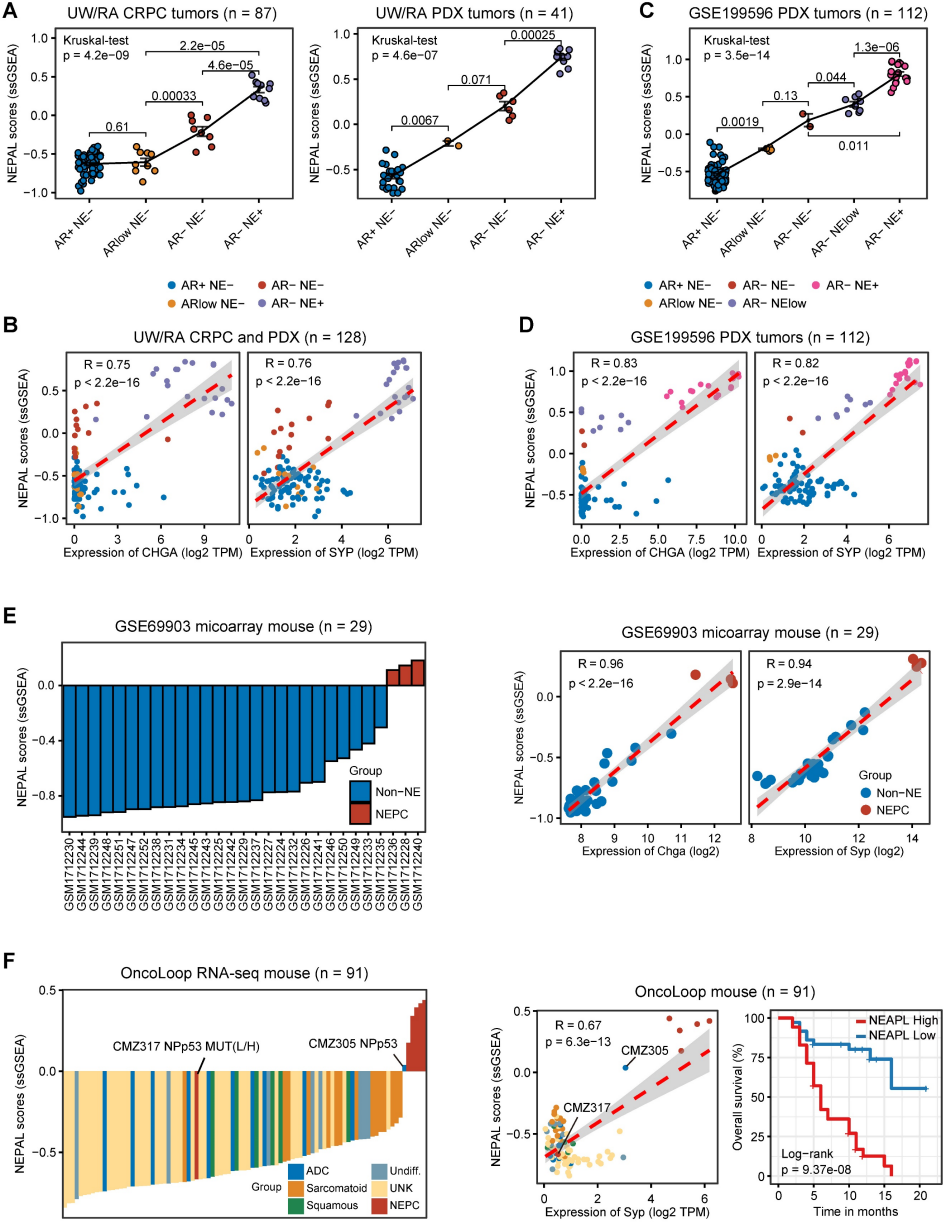
** Application of NEPAL by human PDX datasets and mouse models. A.** The distribution of NEPAL risk scores among different NE features in CRPC (left panel, n = 87) and PDX tumors (right panel, n = 41) in UW/RA dataset. Error bar denote SD. **B.** Pearson correlation between NEPAL risk scores and expression of *CHGA* (left panel) or *SYP* (right panel) in UW/RA dataset (total tumors = 128). **C-D.** Similar analysis to GSE199596 PDX tumors (n = 112). **E.** The distribution of NEPAL risk scores among different NE features in GSE69903 mouse microarray data (left panel, n = 29). And the Pearson correlation between NEPAL risk scores and expression of *Chga* or *Syp* (right panel). **F.** Predicting NEPAL risk scores in genetically engineered mice from different background in OncoLoop cohort (left panel, n = 91). Pearson correlation between NEPAL risk indices and expression of *Syp* and Kaplan-Meier OS curves of mice grouped by NEPAL risk scores (right panel).

In the meantime, we applied NEPAL to bulk transcriptomic datasets generated from 2 human PCa PDX tumors and 3 transgenic mouse models of PCa. The PDX database (UW/RA) contains transcriptomic data of 128 human PCa tumors, including 87 CRPC and 41 PDX tumors 35. Strikingly, in both patients and PDX tumors, the NEPAL scores were tightly correlated with the evolution of AR/NE status (Figure 5A). Concordantly, high Pearson correlation coefficients between predicted NEPC risk scores and NE markers were observed (Figure 5B). Similar association was repeatedly detected in an independent PDX cohort 36 (Figure 5C-D). Furthermore, this phenomenon was not restricted to human PCa, as NEPAL showed coherent performance in two mouse PCa datasets GSE69903 37 (Figure 5E) and GSE90891 12 (Figure S3C). Lastly, using a recent RNA-seq dataset of mouse PCa models 38, NEPAL again exhibited superior accuracy in predicting NEPC status, which led to significant stratification in survival (Figure 5F). These results strengthened the power of NEPAL as an attractive tool to discern NEPC.

### Prognostic value and biological relevance of NEPAL

**Figure 6:**
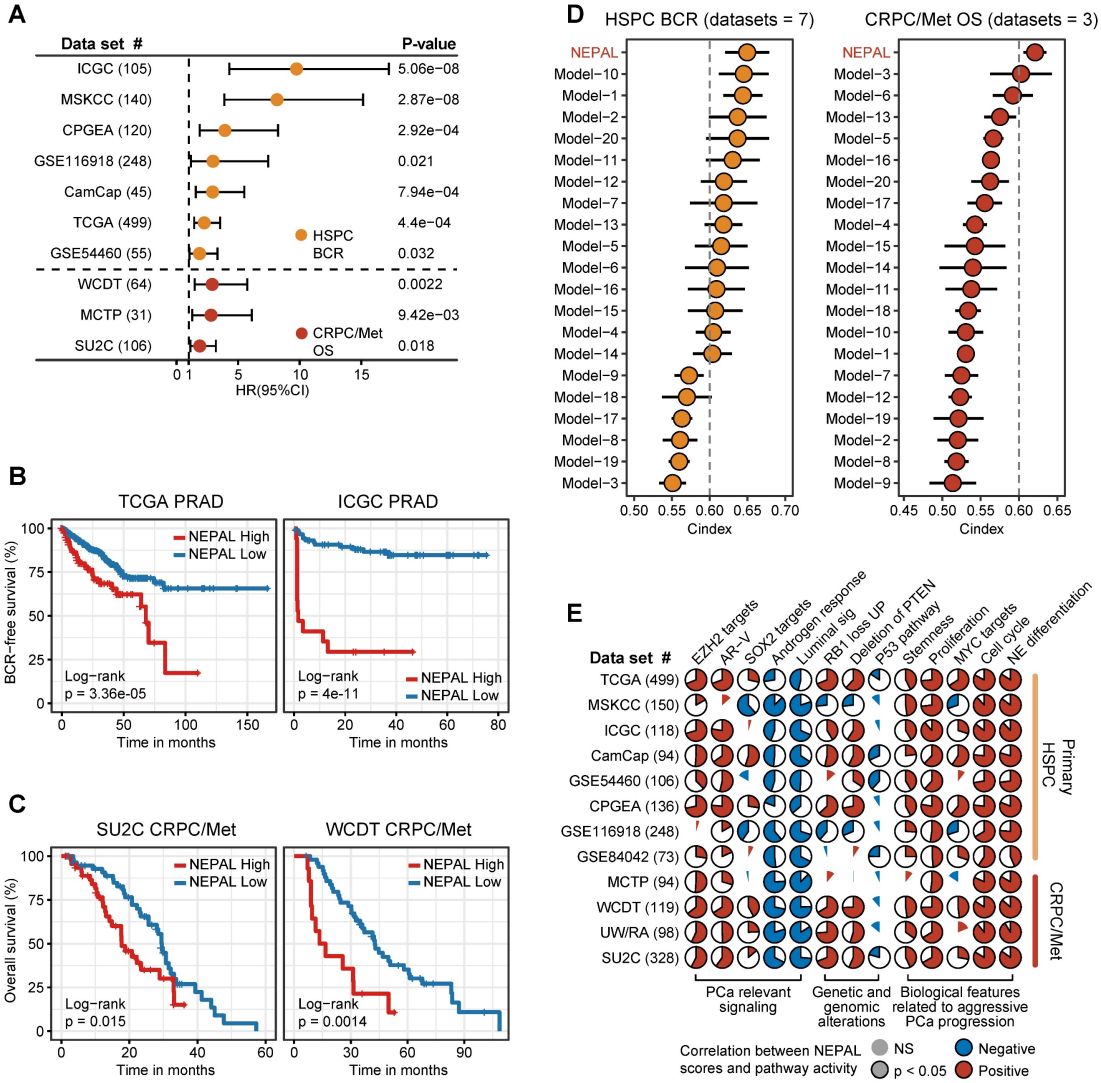
** Prognosis and molecular features associated with NEPAL in human PCa databases. A.** Forest plot for biochemical recurrence (BCR) in primary PCa cohorts or overall survival (OS) in CRPC/Met cohorts. **B-C.** Kaplan-Meier survival curves for BCR (B) or OS (C) in TCGA, ICGC, SU2C or WCDT cohorts. **D.** C-indexes of NEPAL signature and 20 published machine learning prognostic models in 7 primary HSPC datasets (including ICGC, MSKCC, CPGEA, GSE116918, CamCap, TCGA and GSE54460, left panel) and 3 CRPC/Met datasets (including WCDT, MCTP and SU2C, right panel). Error bar denote SD. **E.** Correlation analysis of NEPAL risk scores with activities of multiple signaling pathways in 12 bulk transcriptomic cohorts.

To assess the prognostic value of our NEPAL model, we gathered 12 independent bulk transcriptomic datasets (8 primary HSPC cohorts and 4 CRPC/Met cohorts) with more than 2000 human PCa samples, of which 10 were with available prognostic information. Notably, we observed that NEPAL effectively categorized biochemical recurrence (BCR) for HSPC and overall survival (OS) for advanced PCa patients (Figure 6A-C). In addition, according to the available treatment information, including hormonal therapy, chemotherapy, and second-generation AR signaling inhibition (ARSI) 5, 26, NEPAL also reliably predicted resistance to chemotherapy and ARSI (Figure S4A). Moreover, no significant difference in NEPAL scores was observed between treatment-naïve and -exposed groups in SU2C, UM/SPORE, MCTP, or CPGEA cohorts (Figure S4B-D), suggesting that prior treatment history of patients had little impact on the prognostic accuracy of the NEPAL model. To further compare the prognostic power of our NEPAL model, we collected 20 published prognosis models which are all generated by different machine learning algorithms (Table S2), while also included traditional clinical parameters such as PSA score, Gleason score, and tumor stage. The C-index showed that NEPAL was the most powerful signature than other models and traditional clinical parameters in 10 multicenter PCa cohorts (Figure 6D and Figure S4E), revealing the robustness of NEPAL model in prognostic prediction.

In the meantime, we observed marked correlation of NEPAL risk scores with the activity of lineage plasticity related pathways 2, 12, 46, such as EZH2, SOX2, NE differentiation, as well as loss of RB1, PTEN, and TP53 signaling in all datasets (Figure 6E). Furthermore, NEPAL risk scores were also significantly associated with several hallmarks of advanced PCa 46-48, namely AR-V, cell cycle progression, MYC targets, proliferation and stemness, whereas conversely linked to androgen response and luminal features (Figure 6E). Together, these results potentiated NEPAL to predicting prognosis, therapeutic responsiveness, and molecular characteristics of PCa patients.

### The impact of TME components, patient demographics and tumor stage on the prediction accuracy of NEPAL

To evaluate potential biases introduced by TME components, patient age and race, as well as tumor stage on the prediction accuracy of the NEPAL model, we conducted stratification analyses for these factors. Using three TME component indices including stromal score, immune score and tumor purity 42, our results showed that the NEPAL model maintained its robust predictive capability for patient outcomes (Figure S4F) and NEPC risk (Figure S4G) across diverse TME groups. Meanwhile, the NEPAL model effectively distinguished tumors with NE features in various subtypes of PCa (Figure S4H). Moreover, stratification analysis based on patient age, race, and cancer stage showed that the predictive power of NEPAL model for patient prognosis was not affected by these demographic and pathological factors (Figure S5A-H). Taken together, these results enhance the generalizability and effectiveness of the NEPAL model in predicting NEPC risk and progression.

### NEPAL reveals nongenetic drivers of NEPC

NEPC emergence and progression has been attributed to both genetic and nongenetic factors 4, 14. Along this line, we first stratified the tumors of TCGA PRAD and SU2C CRPC/Met cohorts according to NEPAL score, and analyzed their expression profiles and somatic mutation (Figure S6A-B). Interestingly, of the most frequently mutated genes in PCa, only *TP53* showed higher mutation rate in the NEPC high-risk group compared to the low-risk group in both PRAD and CRPC/Met cohorts, while higher mutation rates of *AR* and *RB1* were observed only in the NEPC high-risk group in SU2C CRPC/Met dataset (Figure S6C-D). Furthermore, both tumor mutational burden (TMB) and mutant counts of all genes were significantly associated with NEPC risk scores in TCGA PRAD dataset, with no significance in SU2C CRPC/Met dataset (Figure S6C-F).

**Figure 7:**
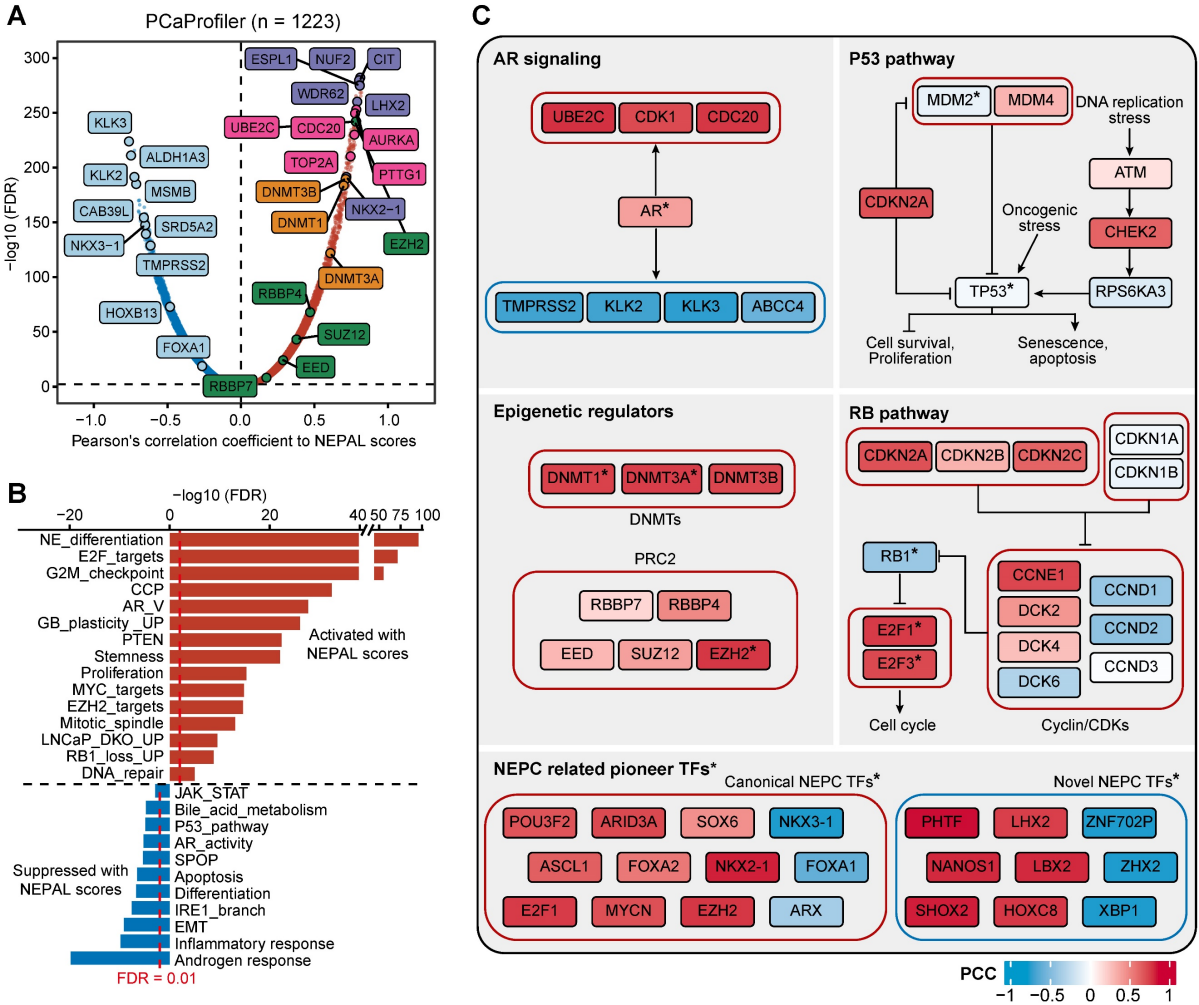
** Predicting NEPC driver genes of nongenetic evolution by NEPAL. A.** Scatter diagram representing the correlation between mRNAs and NEPAL risk scores in PCaProfiler bulk RNA-seq cohort. Positively correlated genes are depicted in red while negatively correlated genes are depicted in blue. NE related markers highlighted in purple, AR signaling related genes in lightcyan, cell cycle related genes in pink, DNA methyltransferases (DNMTs) related genes in orange, and polycomb-repressive complex related genes in green. **B.** GSEA analysis performed on genes ranked for their Pearson's coefficient as determined by the correlation between mRNA expression and NEPAL risk scores. **C.** Comprehensive comparison of gene expression or TFs activity in different NEPC related pathways. * indicates TFs, whose activity is inferred by VIPER method. PCC, Pearson correlation coefficient.

Next, we assessed gene expression correlation with the NEPC risk scores in PCaProfiler with 1223 tissues ranging from normal prostate, primary PCa, CRPC/Met, to NEPC (Figure 7A). In keeping with the lost luminal and acquired NE features during PCa progression 49, canonical NE markers and cell cycle genes were activated, while AR-regulated differentiation genes were suppressed. Importantly, key genes encoding for chromatin remodelers, including DNA methyltransferases (DNMTs), such as *DNMT3A*, *DNMT3B*, *DNMT1*, and members of the polycomb-repressive complex-2 (PRC2), such as *EZH2*, *RBBP4*, *SUZ12*, *EED* and *RBBP7* 12, 29, emerged to the top. This observation was mirrored by identical analyses of the TCGA PRAD and SU2C CRPC/Met datasets (Figure S7A-B), supporting the critical role of epigenetic regulators in NEPC.

Moreover, correlation rank-based GSEA analysis showed that lineage plasticity-related pathways 46, 49-51, such as NE differentiation, glioblastoma (GB) plasticity, loss of PTEN, EZH2 signaling, and dual knockout of RB1 and TP53 up-regulated signaling (LNCaP_DKO_UP), as well as pathways implicated in proliferation and stemness, such as E2F targets, G2M checkpoint, and MYC signaling, were the most significantly activated pathways. On the other hand, pathways related to HSPC, such as androgen response, IRE1α-XBP1s signaling 47, loss of SPOP and AR signaling were suppressed (Figure 7B).

Lastly, combining TF activity inference by VIPER method, we respectively depicted the signaling network of four pathways implicated in NE trans-differentiation, including AR signaling, P53 and RB1 pathways, and epigenetic regulation (Figure 7C). In addition, NEAPL combined VIPER algorithm identified NEPC-related pioneer TFs, including previously established TFs, such as FOXA2, ASCL1 and MYCN 3, 9, 10, as well as novel TFs, such as XBP1s, PHTF, LHX2, and NANOS1 (Table S5). Whether and how these TFs, alone or in cooperation, drive NEPC progression would be of interest for future investigation.

### NEPAL: a computing framework to predict NEPC risk score using transcriptomic data

To facilitate the application of users, we present an R package, NEPAL, which integrates published NE gene sets tested in this study, ssGSEA algorithm for bulk transcriptomic data, AUCell algorithm for scRNA-seq data, multiple machine learning models, and data visualization. In addition, NEPAL supports both mouse and human transcriptomic data as input. We also demonstrate that this workflow shows good compatibility with Seurat scRNA-seq toolkit and is broadly applicable to scRNA-seq datasets based on different platforms. The R package of NEPAL is now available at Github (https://github.com/Famingzhao/NEPAL).

## Discussion

Precise and early identification of NE tumor cells is of great importance for both basic research and clinical practice. Unfortunately, our means in this regard has been constrained by the lack of sensitive and specific biomarkers. To fill in this gap, we designed an integrative analysis pipeline combining bulk and single cell transcriptomic datasets, based on which we identified a high-quality NE gene set and constructed a powerful NEPC risk prediction model with remarkable prognostic power.

Recently, several large-scale comprehensive studies have greatly advanced our understanding of the molecular complexity of PCa microenvironment 4, 5, 9-12, 15-20. Based on these findings, a total of 11 NE gene sets have been proposed to identify NEPC or delineate NE feature thus far. Although several canonical NE markers 8, 9, such as *CHGA*, *CHGB* and *SYP*, are uniformly included, these gene sets displayed considerable inconsistency in genes enrolled, and more importantly, low efficiency in NEPC recognition. One major reason may be that these gene sets heavily depend on bulk transcriptomic data, which is unable to pinpoint NE tumor cells out of the highly heterogeneous TME 15. Fortunately, the challenge can be effectively addressed using emerging techniques, like scRNA-seq. Therefore, we assembled a large scRNA-seq atlas of PCa and performed comprehensive gene expression analysis of these datasets to derive high-quality NEPC-specific gene markers. Such integrative procedures could well balance the strengths and weaknesses of the bulk and scRNA-seq technologies, and allows to identify stable intrinsic gene signature for NE tumor cells. Considering that overfitting during model training has hindered the application and clinical translation, we constructed NEPC risk prediction models using multiple machine learning algorithms, and validated them in 6 independent cohorts by evaluating multiple indexes. Finally, the NE_UP_DN model, NEPAL, was selected with significantly improved performance compared to all previous NEPC gene sets.

Next, we showed that when applied to multiple scRNA-seq datasets, NEPAL could depict the path of NEPC cell evolution in 8 NEPC-representative subclusters. Exploration across various human HSPC datasets provided further evidence that NEPAL might serve as a powerful tool to describe the roadmap of PCa progression. In addition, NEPAL had potential application value in a variety of experimental models of PCa, including human PCa cell lines and PDX, as well as transgenic mouse models of PCa. For example, in two PDX models, we noted that NEPAL scores were strongly associated with distinct stages of PCa, from hormone-naïve, AR-negative, to NE-positive late-stage disease. These results demonstrate that NEPAL may be applied to portrait PCa progression of both human and mouse origins, based on gene expression profiles of both bulk and single-cell sources.

Previous work has assessed at least 10 prognosis models derived by different gene signatures and machine learning algorithms, which generally performed poorly in survival prediction (AUC < 0.6) 25. On the contrary, and to our surprise, the NEPAL signature exhibited exciting power in predicting not only prognosis, such as BCR and OS, but also resistance to hormonal therapy and chemotherapy in more than 10 human PCa datasets. Furthermore, compared to 20 published prognostic models of PCa as well as existing clinical parameters, our NEPAL demonstrated robust and superior predictive capacity. Of note, factors including patient demographic, cancer stage, PCa subtype, and treatment history showed little to no impact on the predictive power of the NEPAL model, which increased its generalizability and effectiveness. Regarding the molecular characteristics, NEPAL risk scores were closely associated with several pathways related to aggressive PCa progression, such as AR-V, proliferation and stemness 46-48. In addition, patients with loss of *TP53* or *RB1*, a critical driver of lineage plasticity and therapeutic resistance 12, 46, 51, had a higher NEPC risk scores than their wild-type counterparts. Taken together, these results implicate that NEPAL can be applied as a prognostic evaluation tool for PCa.

Taking advantage of NEPAL, we were also able to identify critical drivers of NEPC, especially the nongenetic ones, for instance, PRC2 and DNMTs. These observations are in line with the notion that on top of the oncogenic drivers, marked epigenetic changes, made by the PRC2 and DNMTs for instance, further facilitate NE lineage progression 3. Furthermore, we compared these drivers in the canonical pathways, such as AR, TP53 and RB1 pathways. Moreover, we identified a series of TFs related to NEPC, including both well-established NEPC pioneer TFs, and novel TFs, such as XBP1s. Our previous studies have unveiled the signaling network between the AR pathway, the IRE1α-XBP1s branch of the unfolded protein response, and the c-Myc oncogenic program in promoting PCa 47, 48. But whether and how the IRE1α-XBP1s pathway facilitates the transition to NEPC requires functional and mechanistic investigation. Nevertheless, these results manifest that NEPAL captures key molecular events during NEPC progression and may offer new insights for future characterization of NEPC.

## Conclusion

We constructed and extensively validated a robust NEPC risk prediction model NEPAL, which is a competent tool for precision identification of NEPC for basic research purposes, and lays a solid foundation for future clinical translation.

## Supplementary Material

Supplementary figures.

Supplementary tables.
