# Supplementary material for: Integrated analysis of single-cell and bulk transcriptomics develops a robust neuroendocrine cell-intrinsic signature to predict prostate cancer progression
Source: Theranostics. 2024 Jan 1;14(3):1065–80. doi: 10.7150/thno.92336 (PMC10797290; doi:10.7150/thno.92336)
Supplement: Supplementary file 1 — Supplementary figures. [file thnov14p1065s1.pdf]

Integrated analysis of single-cell and bulk transcriptomics develops a robust neuroendocrine cell-intrinsic signature to predict prostate cancer progression

Tingting Zhang[1,2#], Faming Zhao[1,2#]*, Yahang Lin[3], Mingsheng Liu[4], Hongqing Zhou[4], Fengzhen Cui[1,2], Yang Jin[5], Liang Chen[6], Xia Sheng[1,2]*

[1]Key Laboratory of Environmental Health, Ministry of Education & Ministry of Environmental Protection, School of Public Health, Tongji Medical College, Huazhong University of Science and Technology, Wuhan, China.

[2]School of Life and Health Sciences, Hainan University, Haikou, China.

[3]Department of Neurology, Wuhan Fourth Hospital/Pu'ai Hospital, Wuhan, China.

[4]The Second Ward of Urology, Qujing Affiliated Hospital of Kunming Medical University, Qujing, China.

[5]Institute for Cancer Genetics and Informatics, Oslo University Hospital, Oslo, Norway.

[6]Department of Urology, Tongji Hospital, Tongji Medical College, Huazhong University of Science and Technology, Wuhan, China.

[#]These authors contributed equally to this work.

*Corresponding authors: Faming Zhao (famingzhao@hust.edu.cn) and Xia Sheng (xiasheng@hust.edu.cn).

Keywords: neuroendocrine prostate cancer, single-cell, biomarker, computational biology and bioinformatics, machine learning

Supplemental Figures

Figure S1

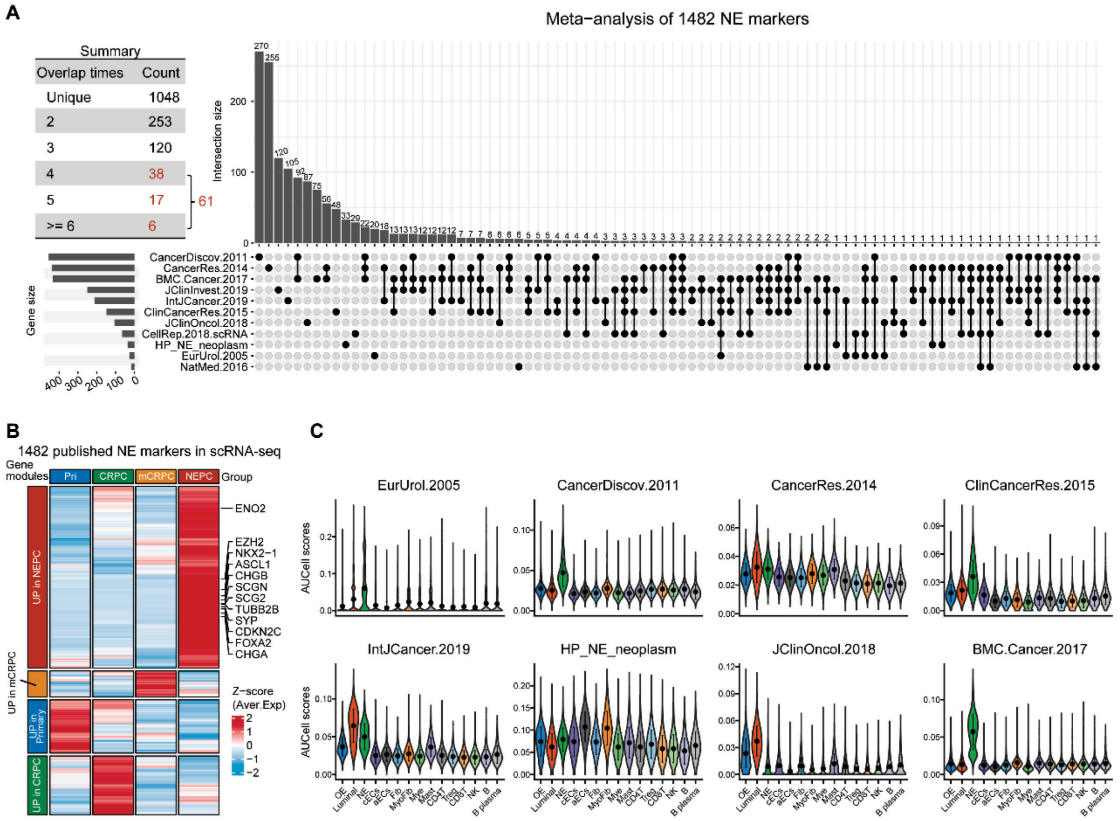

Figure S1. NE meta-gene sets comprise a total of 1482 genes with low overlap rate.

A. Upset plot showing the intersection of 11 literature NE gene-lists.

B. Heatmap showing the expression (Z-score) of 1482 published NE markers in different tumor types.

C. AUCell enrichment analysis comparing different NE gene sets in each cell type.

Figure S2

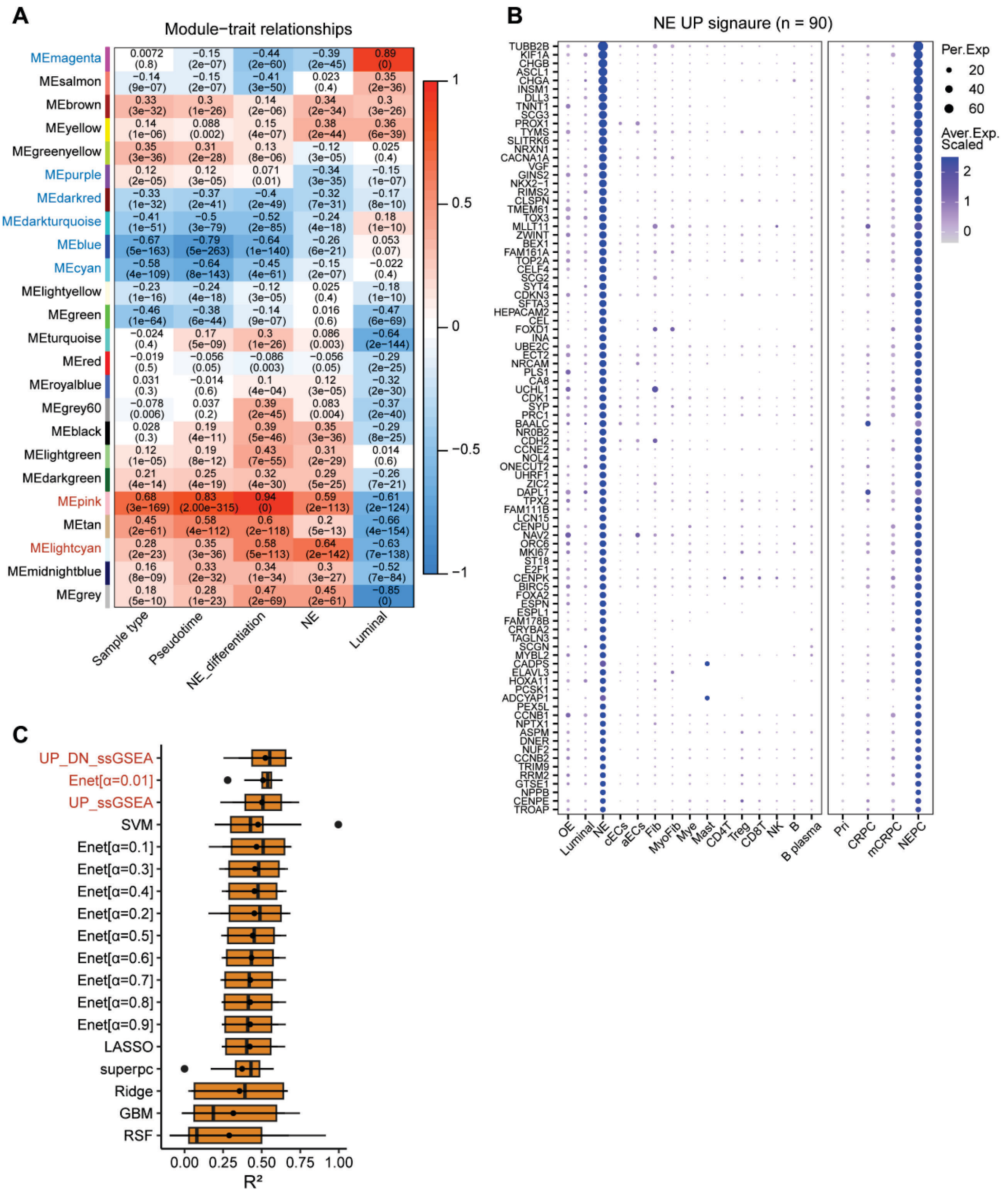

Figure S2. Combining multiple strategies to identify NEPC feature genes based on scRNA-seq and bulk RNA-seq meta-databases.

A. Correlation analysis between module eigengenes and clinical traits by WGCNA analysis.

B. Dot plot of NE_UP signature genes (n = 90) identified by this study for each cell

cluster and tumor group.

C. The average R^2 index of 18 algorithms in the 6 testing cohorts. Error bar denote SD.

Figure S3

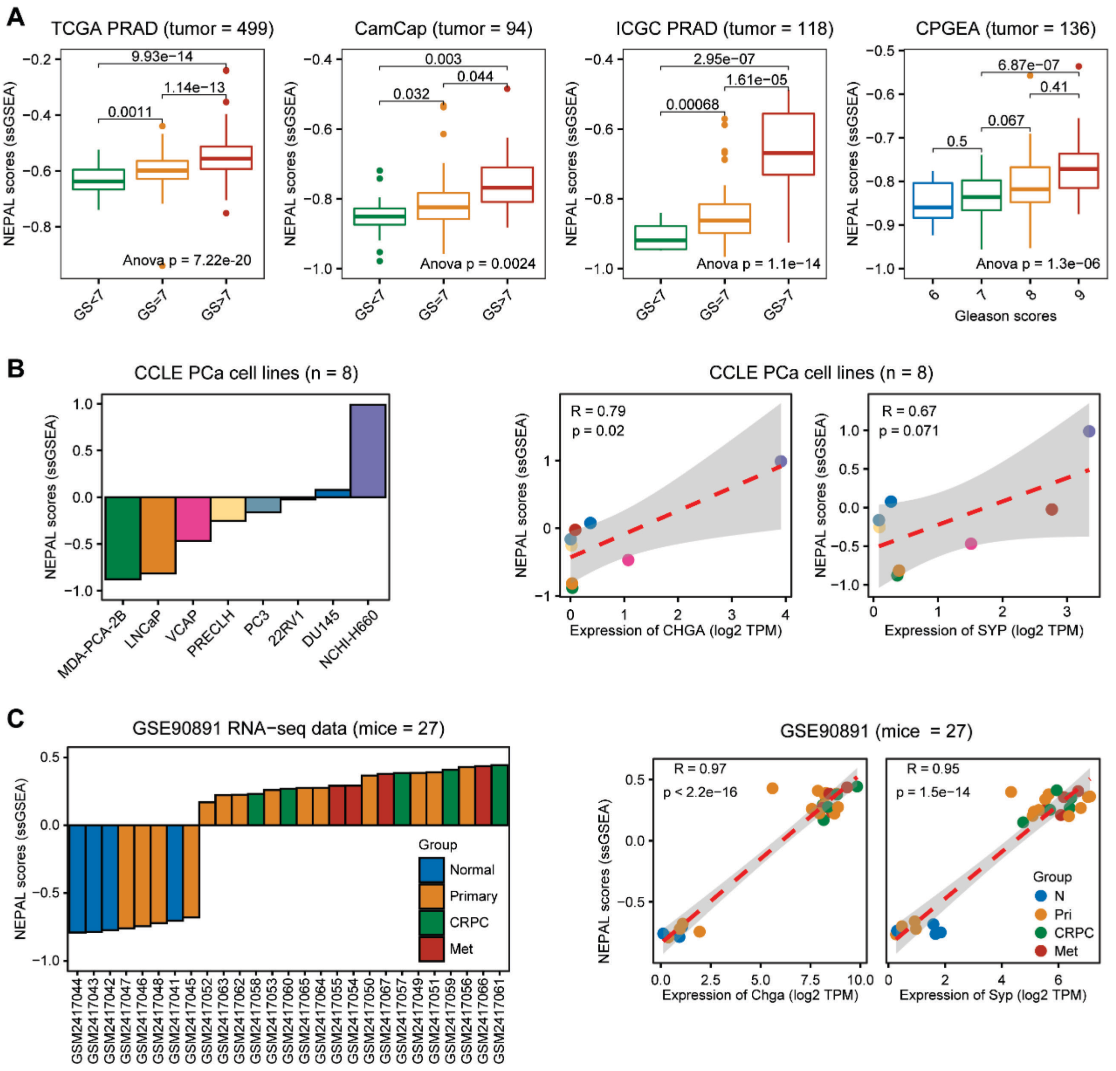

Figure S3. Validation of NEPAL risk model in human, cell lines and mouse transcriptomic data.

A The distribution of NEPAL risk scores among different Gleason score groups in TCGA, CamCap, ICGC and CPGEA human PCa cohorts. GS, Gleason scores. The box represents the interquartile range, the horizontal line in box is the median, and the whiskers represent 1.5 times interquartile range.

B. Predicting NEPAL risk scores for 8 PCa cell lines from CCLE database (left panel). Pearson correlation between NEPAL risk scores and expression of *CHGA* or *SYP* in

PCa cell lines (right panel).

C. Similar analysis to GSE90891 RNA-seq data of mice with PCa (n = 27).

Figure S4

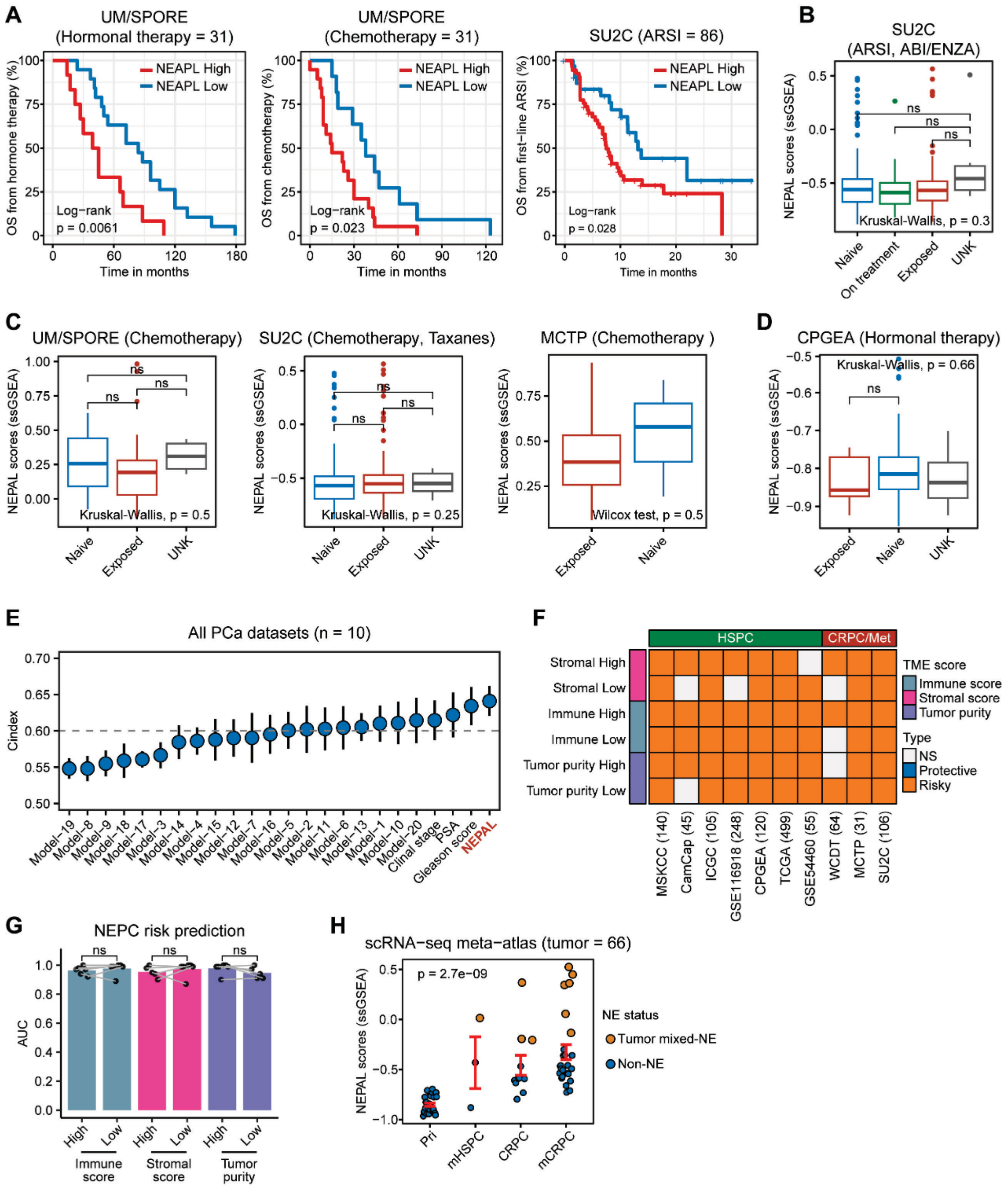

Figure S4. Assessment the effects of prior treatment history, TME components and various subtypes on the prediction accuracy of the NEPAL model.

A. Therapeutic resistance analysis by Kaplan–Meier OS curves of patients grouped by NEPAL risk scores.

B-D. Box plots showing the distribution of NEPAL scores among different ARSI (B),

chemotherapy (C), or hormonal therapy groups (D) in corresponding cohorts. The box represents the interquartile range, the horizontal line in box is the median, and the whiskers represent 1.5 times interquartile range.

E. C-indexes of NEPAL signature, 20 published machine learning prognostic models, and traditional clinical parameters across 10 multicentric PCa cohorts. These cohorts include 7 primary HSPC datasets (ICGC, MSKCC, CPGEA, GSE116918, CamCap, TCGA and GSE54460), as well as 3 CRPC/Met datasets (WCDT, MCTP and SU2C).

F-G. The stratification survival analyses between groups with high and low TME scores to assess the effectiveness of the NEPAL score in predicting PCa progression (F) and NEPC risk (G). Each dot represents an individual data sets.

H. The distribution of NEPAL scores among the various subtypes of PCa in the scRNA-seq meta-atlas. Each dot represents an individual sample. Tumors without NE features depicted in blue. Tumors with NE features depicted in yellow.

Error bar denote SD (E, G and H).

Figure S5

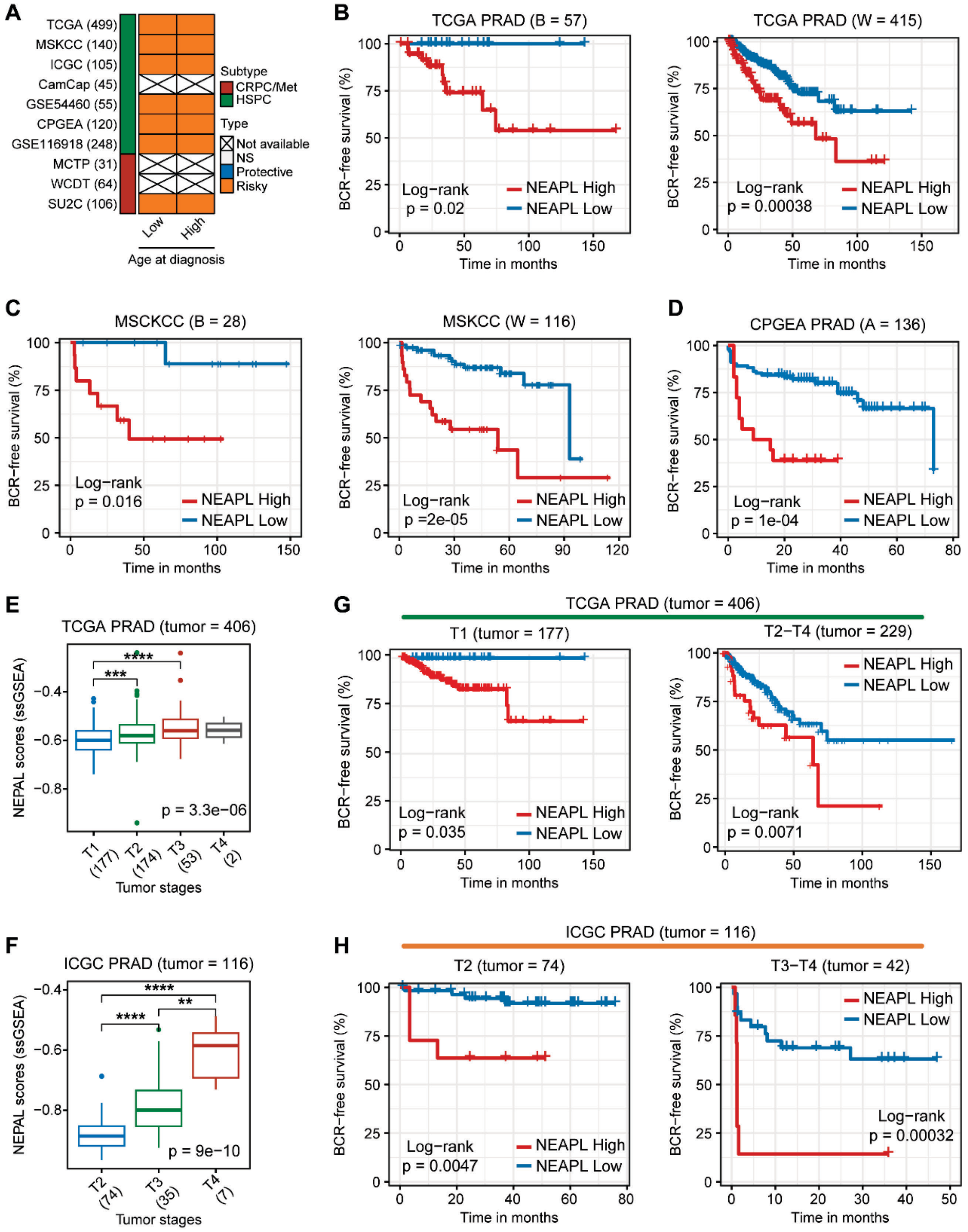

Figure S5. Assessment the impact of patient age, race and tumor stages on the prediction accuracy of the NEPAL model.

A. The stratification survival analyses based on patient age at diagnosis to assess the effectiveness of the NEPAL score in predicting PCa progression.

B-D. The stratification analysis of patient race showing the outcomes for patient groups with low and high NEPAL scores in the TCGA PRAD cohorts. B, Black or African American; W, White; A, Asian.

E-F. The distribution of NEPAL scores among patient groups with different tumor stages in TCGA (E) and ICGC (F) PRAD cohorts.

G-H. The stratification analysis of tumor stages showing the outcomes for patient groups with low and high NEPAL scores in the TCGA (G) and ICGC (H) cohorts.

Figure S6

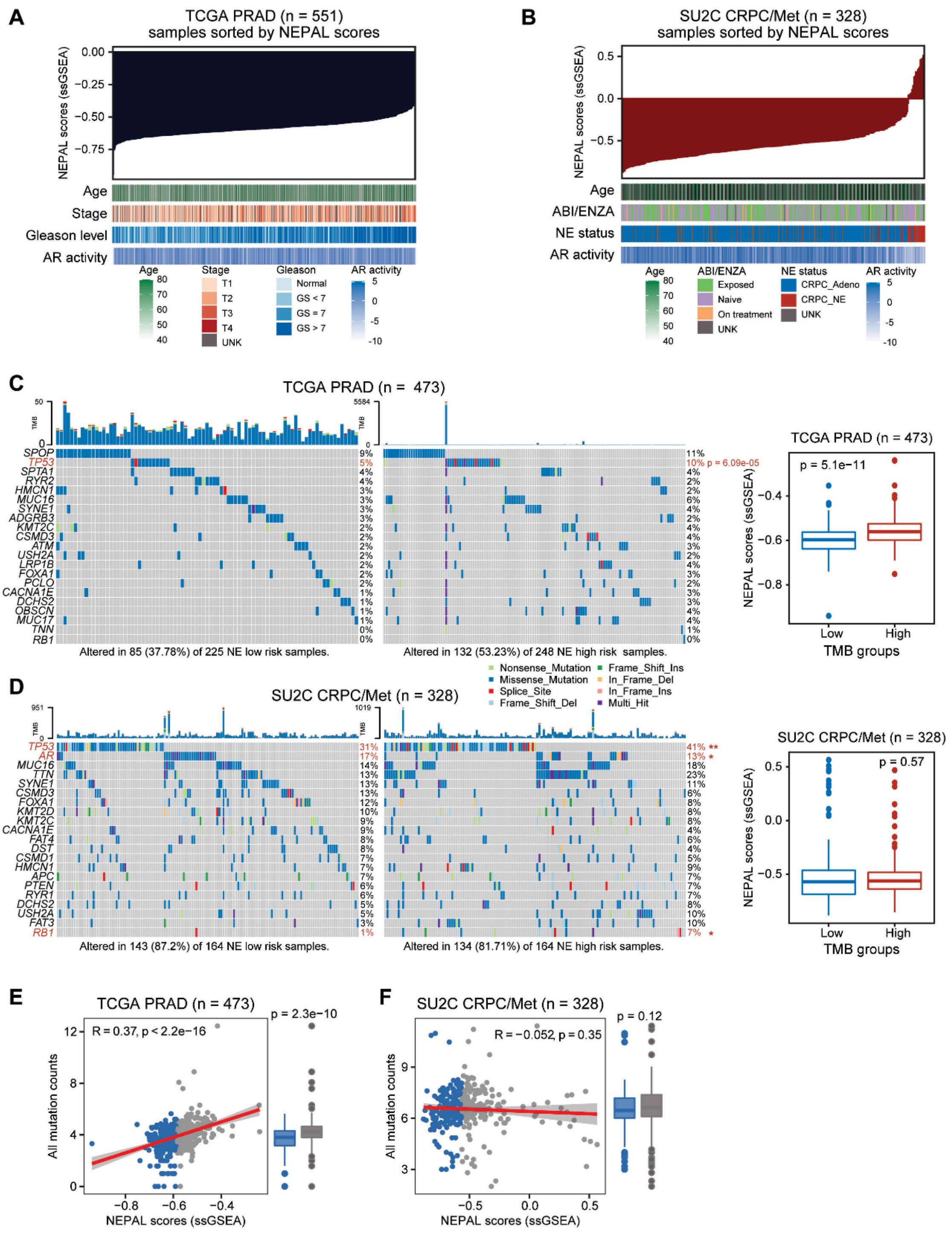

Figure S6. Associations between the NEPAL risk scores and genetic alterations in human PCa databases.

A-B. An overview of the association between known clinical features and NEPAL risk

scores in TCGA PRAD (n = 551, A) and SU2C CRPC/Met (n = 328, B) databases. Columns represent samples sorted by NEPAL scores from low to high (top row). Rows represent known clinical features. GS, Gleason scores.

C. Top 21 highly mutated genes in low- and high- NEPAL risk score groups from TCGA PRAD tumors (left panel). NEPAL risk scores of different tumor mutational burden (TMB) high- and low-groups (right panel).

D. Similar analysis to SU2C CRPC/Met cohort (n = 328). *, $p < 0.05$; **, $p < 0.01$.

E-F. Correlation analysis between NEPAL risk scores and all gene mutation counts in TCGA PRAD (E) and SU2C CRPC/Met (F) cohorts. Bule representing patients with low NEPAL risk scores. Gray representing patients with high NEPAL risk scores.

Figure S7

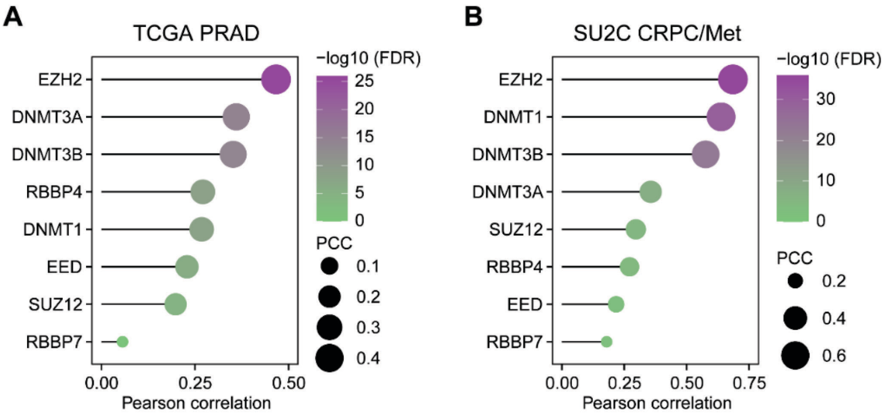

Figure S7. Identification nongenetic evolution drivers for NEPC.

A-B. Pearson correlation analysis between NEPAL risk scores and indicated genes in TCGA PRAD (A) and SU2C CRPC/Met (B) cohorts. PCC, Pearson correlation coefficient.

Supplemental Tables

Supplemental table 1. Cohorts and cell type markers for the scRNA-seq data used in this study.

Supplemental table 2. List of published NEPC_Meta gene signatures and prognostic machine learning models for PCa.

Supplemental table 3. NEPC markers and signature gene-lists in the scRNA-seq meta-atlas.

Supplemental table 4. The predicting results of NEPC risk scores using multiple models across six PCa cohorts.

Supplemental table 5. The correlation between gene expression or transcription

factors activities and the NEPAL scores in PCaProfiler dataset.
